# Olive Mill Wastewater as Source of Polyphenols with Nutraceutical Properties

**DOI:** 10.3390/nu15173746

**Published:** 2023-08-26

**Authors:** Doretta Cuffaro, Andrea Bertolini, Simone Bertini, Claudio Ricci, Maria Grazia Cascone, Serena Danti, Alessandro Saba, Marco Macchia, Maria Digiacomo

**Affiliations:** 1 Department of Pharmacy, University of Pisa, Via Bonanno 6, 56126 Pisa, Italy; doretta.cuffaro@unipi.it (D.C.); simone.bertini@unipi.it (S.B.); marco.macchia@unipi.it (M.M.); 2 Interdepartmental Research Center “Nutraceuticals and Food for Health”, University of Pisa, 56100 Pisa, Italy; alessandro.saba@unipi.it; 3 Department of Surgery, Medical, Molecular and Critical Area Pathology, University of Pisa, 56126 Pisa, Italy; a.bertolini2@student.unisi.it; 4 Department of Civil and Industrial Engineering, University of Pisa, 56122 Pisa, Italy; claudio.ricci@unipi.it (C.R.); maria.grazia.cascone@unipi.it (M.G.C.); serena.danti@unipi.it (S.D.); 5 Department of Translational Research and New Technologies in Medicine and Surgery, University of Pisa, 56126 Pisa, Italy

**Keywords:** olive mill wastewater, extra virgin olive oil, nutraceuticals, antioxidants, anti-inflammatory properties

## Abstract

Background: Agrifood waste products are often considered rich sources of bioactive compounds that can be conveniently recovered. Due to these peculiar characteristics, the study of these waste products is attracting great interest in nutraceutical research. Olive mill wastewaters (OMWWs) are generated by extra virgin olive oil (EVOO) production, and they pose environmental challenges due to their disposal. This study aimed to characterize the polyphenolic profile and to evaluate the nutraceutical properties of OMWW extracts from two Tuscan olive cultivars, Leccino (CL) and Frantoio (CF), collected during different time points in EVOO production. Method: After a liquid–liquid extraction, the HPLC and LC–MS/MS analysis of OMWW extracts confirmed the presence of 18 polyphenolic compounds. Results: The polyphenol composition varied between the cultivars and during maturation stages. Notably, oleacein was detected at remarkably high levels in CL1 and CF1 extracts (314.628 ± 19.535 and 227.273 ± 3.974 μg/mg, respectively). All samples demonstrated scavenging effects on free radicals (DPPH and ABTS assays) and an anti-inflammatory potential by inhibiting cyclooxygenase (COX) enzymes. Conclusions: This study highlights the nutraceutical potential of OMWW extracts, emphasizing their antioxidant, antiradical, and anti-inflammatory activities. The results demonstrate the influence of olive cultivar, maturation stage, and extraction process on the polyphenolic composition and the bioactivity of OMWW extracts. These findings support a more profitable reuse of OMWW as an innovative, renewable, and low-cost source of dietary polyphenols with potential applications as functional ingredients in the development of dietary supplements, as well as in the pharmaceutical and cosmetics industries.

## 1. Introduction

Extra virgin olive oil (EVOO) contributes to the health benefits of the Mediterranean diet, being the main source of fats and displaying numerous nutraceutical properties [1,2]. EVOO production usually occurs in a specific period, i.e., from September to December, though depending on the area and on the pedoclimatic conditions. It consists of the mechanical extraction of olives and includes several steps: leaf removal and washing, crushing, mixing or malaxation, and, finally, the extraction process. The modern EVOO extraction techniques involve two different processes: two- and three-phase decanter extraction. The two-phase system does not require the further addition of water, resulting in olive oil and a semi-solid olive cake. The three-phase decanter process requires the addition of hot water and results in olive oil, olive mill wastewater (OMWW), and olive pomace. Through the three-phase process, during the extraction of EVOO, a huge volume of OMWW is generated, considering that the usual ratio of olive oil production to OMWW is 1.0:2.5 L and that, in Italy, 1.4 million m^3^ of OMWW is produced, as well as 30 million m^3^ in the Mediterranean basin [3]. OMWW is characterized by marked acidity and a high content of phenolic components, and due to these peculiarities, it can potentially be toxic to the air, microorganisms, and plants [4]. Therefore, OMWW disposal constitutes an environmental concern with high-costing procedures, and correct management represents a challenge. Nowadays, a great effort is being directed towards the reuse of food-chain wastes in terms of sustainability and revalorization. 

Generally, the disposal of OMWWs in waterways or in soil still represents a serious issue due to their phytotoxicity and antimicrobial properties that can affect the balance of ecological systems. In many cases, direct disposal of OMWWs into water streams or in irrigation has resulted in environmental consequences due to the OMWW-high content of phenolic compounds, toxic elements for plants, resulting in deleterious effects on the soil [5,6,7,8]. Despite this, polyphenols are widely recognized as natural bioactive molecules with important nutraceutical properties. Therefore, the recovery of phenolic compounds from OMWWs not only provides an economic opportunity but also decreases the environmental impact [5]. In fact, phenolic compounds present in OMWW display well-known biological effects and health benefits, which should be valorized [9].

As has emerged from the literature, the main phenolic compounds present in OMWW are hydroxytyrosol, tyrosol, verbascoside, and cinnamic acids, such as caffeic, gallic, vanillic and syringic acids [10,11,12]. Recently, researchers aimed to recover polyphenols from OMWW for possible use in the food industry, mainly as nutraceuticals [13,14]. Nowadays, the recovery of valuable bioactive polyphenols from food supply chain by-products is of great interest in terms of sustainability and revalorization. It was demonstrated that phenolic compounds extracted from OMWW exhibit anti-inflammatory action, as they are able to decrease the production of nitric oxide (NO) in LPS-stimulated cells [15]. Moreover, OMWW extracts display in vitro and in vivo antiangiogenic and chemo-preventive capacities [16], inhibiting the proliferation, migration, and invasion of endothelial cells [17]. Due to the interesting properties of OMWW polyphenols, recently, different applications of OMWW extracts in cosmetic fields [18] or in food applications [19] have been successfully described. Moreover, the polyphenol-rich OMWW extracts could be applied as active ingredients in the development of dietary supplements or food products as added value in the formulation, presenting new functionalities such as enhanced nutritional properties. 

Considering that OMWW composition primarily depends on the extraction system and olive cultivar, in this work, we studied several samples of OMWWs generated by a three-phases EVOO extraction system. In particular, we analyzed OMWWs produced by two different cultivars of olives: cultivar Leccino (CL) and Frantoio (CF). The collection was conducted in October (CF1 and CL1) and November (CF2 and CL2) in order to investigate the variations of polyphenols content over the time. The OMWW extracts have been obtained via liquid–liquid extraction, allowing for the recovery of important dietary polyphenols. The polyphenolic profile of OMWW extracts was defined by the analytical quantification performed by LC–MS/MS analysis, including the identification and quantification of 18 polyphenols. Furthermore, with the aim to valorize the polyphenolic extract obtained by these waste products and to highlight their nutraceutical properties, the antioxidant profile, and the anti-inflammatory effect in terms of cyclooxygenases (COX 1 and COX2) inhibition were evaluated. This study well-aligns with the purposes of environmental sustainability, exploiting the recovery of valuable bioactive polyphenols from food supply chain by-products such as OMWW.

## 2. Materials and Methods

### 2.1. Chemistry

Solvents used for extraction procedures and high-performance liquid chromatography (HPLC) coupled to tandem mass spectrometry (LC–MS/MS) analyses, such as LC–MS-grade and HPLC-grade water, LC–MS- and HPLC-grade methanol (MeOH), LC–MS- and HPLC-grade acetonitrile (ACN), LC–MS-grade 2-propanol, HPLC-grade *n*-hexane, HPLC-grade ethanol (EtOH), HPLC-grade ethyl acetate, HPLC-grade acetic acid (AcOH, 100%), and LC–MS-grade formic acid (FA, ≥8%), were all purchased from Sigma Aldrich-Merck (Merck srl, Milan, Italy). The pure standards of oleocanthal, oleocanthalic acid, and oleacein were obtained via EVOO extraction and purification using the method developed in our previous study [20,21]. The commercial analytical standards of pinoresinol, *p*-hydroxyphenylacetic acid, oleouropein, ibuprofen, and Trolox were purchased from Merck (Merck srl, Milan, Italy); tyrosol, hydroxytyrosol, caffeic acid, vanillic acid, ferulic acid, *p*-coumaric acid, syringic acid, and vanillin were purchased from TCI (Zwijndrecht, Belgium); the commercial analytical standards luteolin-7-glucoside, apigenin-7-glucoside, verbascoside, and rutin were purchased from TRC (Toronto chemicals). 1-acetoxypinoresinol was identified after isolation via semipreparative HPLC and confirmed via LC–MS/MS analysis and quantified using the Pinoresinol calibration curve as already reported [22]. 2,2-Diphenyl-1-picrylhydrazyl (DPPH) and 2,4,6-Tris(2-pyridyl)-s-triazine (TPTZ) and (ABTS) were purchased from Merck (Darmstadt, Germany).

### 2.2. Extraction of OMWW

OMWW samples belong to two different cultivars: Cultivar Leccino (CL) and Cultivar Frantoio (CF). The samples were collected twice during EVOO production: October 2022 (CL1 and CF1) and November 2022 (CL2 and CF2). After direct collection from the decanter, samples were frozen and stored at −20 °C. Liquid–liquid extraction was carried out following the procedure reported by De Marco et al. [23]. Briefly, 10 mL of OMWW was acidified to pH 2 with HCl 1N and washed with *n*-hexane (15 mL) in order to remove the lipid fraction. The mixture was vigorously shaken and centrifuged for 5 min at 3000 rpm. The phases were separated, and the step was repeated successively two times with *n*-hexane. Extraction of phenolic compounds was then carried out with 10 mL of ethyl acetate. The mixture was vigorously shaken and centrifuged for 5 min at 3000 rpm. The phases were separated, and the extraction was repeated successively three times. The ethyl acetate was evaporated under reduced pressure, obtaining the solvent-free dry residue. 

### 2.3. LC–MS/MS Instrumental Layout and Parameters

The instrument layout consisted in an Agilent (Santa Clara, CA, USA) 1290 UHPLC system, including a binary pump, a column oven set at 40 °C, and a thermostated autosampler, coupled to an AB-Sciex (Concord, Ontario, Canada) QTRAP 6500+ mass spectrometer working as a triple quadrupole, equipped with an IonDrive™ Turbo V source set for electrospray ionization (ESI). Chromatographic separation was achieved by using a 4.6 × 150 mm, 5 μm particle size Agilent Zorbax SB-C18 StableBond Analytical column (Santa Clara, CA, USA), and using (A) MeOH and (B) water (100%), both mixed with 0.025% CH_3_COOH, as mobile phases. Gradient elution was performed at 800 μL/min as follows: 0.0–1.0 min (B) 100%; 12.0–14.0 min (B) 5%; 15.0–18.0 min (B) 100%. The injection volume was set at 10 μL. System control and data acquisition were carried out with ABSciex Analyst^®^ version 1.7 software, data processing was carried out with Multiquant^TM^ software version 3.0.3, while data analysis was performed using Microsoft 365^®^ Excel software version 2307 (Albuquerque, NM, USA) and GraphPad (Boston, MA, USA) Prism version 9.0.2.

A mass spectrometry selected reaction monitoring (SRM) method was operated in negative-ion mode. For each compound, after the optimization of declustering potential (DP), collision energy (CE), and collision exit potential (CxP), three transitions were considered in the analysis. One of them was integrated and used as a quantifier (Q), and the other two were used as qualifiers (q), as reported in Table 1. Further operative parameters were gas source 1 (GS1), 40 arbitrary units; gas source 2 (GS2), 40 arbitrary units; ion spray voltage (ISV), −4.0 kV; source temperature (TEM), 450 °C; Curtain gas (CUR), 20 arbitrary units; and collision gas (CAD) N_2_—operative pressure with CAD gas on, 2.93 mPa.

Standard stock solutions and sample preparation: a stock solution of each standard compound, including the internal standard, was prepared in MeOH at a concentration of 100 μg/mL and stored at −20 °C. Calibration curves were freshly prepared in the range of concentrations reported in Table S1. A mixture of all polyphenols at their higher concentration was prepared and then serially diluted in water. Different starting concentrations for each analyte were optimized in order to achieve linearity in the widest range possible. Each calibration point was mixed with the appropriate amount of the internal standard to achieve the same final concentration (500 ng/mL) in both the submitted samples and in the curve. Since standards were not commercially available, and considering the similar behavior and fragmentation patterns of both 1-acetoxypinoresinol and pinoresinol, the quantitation of 1-acetoxypinoresinol was achieved using an external calibration curve built on the common SRM transition 357.3 → 136.6 and 415.3 → 136.6 (Q). Extracted OMWW samples (10–100 mg), stored at −20 °C, were thawed at room temperature (rt) and resuspended in 1000 μL of a H_2_O:MeOH mixture (50:50, *v/v*). Then, samples were diluted in water at different ratios—1:10, 1:100—1:1000, and 1:20,000— to allow each analyte to be correctly quantified in its relatively built calibration curve, mixed with the appropriate amount of internal standard, and 10 μL were injected into the LC–MS/MS system. All samples were analyzed in triplicate.

### 2.4. DPPH• Radical Scavenging Activity

The antioxidant activity of OMWW extracts (CT1, CT2, CF1, and CF2) was evaluated using the DPPH• free radical scavenging assay as described by Cuffaro et al. [24]. Samples were solubilized in MeOH at different concentrations (from 0.1 mg/mL to 1.5 mg/mL) and added to a methanol solution of DPPH• (40.0 μg/mL). After 45 min of incubation at rt and in the dark, the absorbance was read at 517 nm in a SPECTROstarNano (200–1000 nm) UV/Vis spectrophotometer. MeOH and Trolox^®^ were used as blank and standard positive control, respectively, and were treated under the same conditions as the samples. The percent of antioxidant activity (%AA) was calculated according with the following formula:

%AA = (AbsDPPH − Abssample/AbsDPPH) × 100,

where

AbsDPPH = absorbance of the DPPH solution, subtracted of absorbance of MeOH;

Abssample = absorbance of the DPPH solution containing the test compound subtracted of the absorbance of test compound solution without DPPH. 

The results were expressed as inhibitory concentrations at 50% (IC_50_), calculated via linear regression. All experiments were performed in triplicate.

### 2.5. Ferric Reducing Antioxidant Power Assay (FRAP)

The method described by Borges et al., with some modifications [24,25], was used to assess the antioxidant activity. FRAP assay is based on iron reduction, measuring the ferric-reducing ability of a sample in an acid medium (pH 3.6) through the formation of a specific blue color as the ferric tripyridyltriazine (Fe^3+^–TPTZ) complex due to the reduction to the ferrous (Fe^2+^) form. FRAP reagent was prepared by mixing 0.3 M acetate sodium buffer pH = 3.6, 20 mM ferric chloride, and 10 mM TPTZ in 40 mM HCl at a ratio 10:1:1. Amounts of 20 µL of the extracts were mixed with 280 µL of the FRAP solution, and the mixture was incubated at 37 °C for 30 min. The absorbance of the reaction mixture was read at 595 nm in a SPECTROstarNano (200–1000 nm) UV/Vis spectrophotometer. The calibration curve was built using different concentrations of Trolox (0.01–0.2 mg mL^−1^), and the results are expressed as the mmol of Trolox equivalents per g of the sample. All experiments were conducted in triplicate.

### 2.6. ABTS Assay

The free radical scavenging activity of samples was determined by the ABTS radical cation decolorization assay using the protocol reported by Pellegrini et al. with some modifications [24,26]. Briefly, ABTS solution was prepared by mixing 7 mM of aqueous solution of ABTS with 2.45 mM potassium persulfate in a 1:1 ratio. The solution was incubated for 12 h in the dark at rt; then, it was diluted with EtOH to an absorbance of 0.7 at 750 nm. Then, 180 µL of the ABTS solution were mixed with 10 µL of samples in EtOH, and the solution was incubated 5 min at rt; then, the final absorbance was read at 734 nm. Calculations were performed by evaluating the percentage of inhibition of the ABTS radical cation as follows:

% scavenging ability = (AbsABTS − Abssample/AbsABTS) × 100,

where

AbsABTS = the absorbance of the ABTS solution;

Abssample = the absorbance of the ABTS solution containing the test compound.

The percentage of scavenging ability was calculated against the sample concentration to obtain the inhibitory concentration. All experiments were performed in triplicate.

### 2.7. Cyclooxygenase Enzyme Inhibitory Assay

The ability of CT1, CT2, CF1, and CF2 extracts to inhibit COX-1 and COX-2 was evaluated using a COX-1 (ovine) and COX-2 (human)-inhibitor screening assay (kit No. 701050 from Cayman Chemical Co., Ann Arbor, MI, USA) following the manufacturer’s protocols. COX-1/COX-2 inhibitory evaluation test was performed at 1 mg/mL for all the extracts. Then, increasing concentrations of each sample (0.01–1 mg/mL) were tested. Arachidonic acid at 1.1 mM was the substrate and ibuprofen was used as a control. The peroxidase activity was examined colorimetrically at 590 nm after an incubation of 120 min at rt using a SPECTROstarNano (200–1000 nm) UV/Vis spectrophotometer (BMG Labtec, Germany). All tests were performed three times. The percent (%) inhibition of COX-1 and COX-2 is derived from the following formula:

% inhibition = (EAA − AIA)/EAA × 100,

where

EAA = Enzyme test activity absorbance;

AIA = Activity inhibition test absorbance.

Results were expressed as percent (%) inhibition or inhibitory concentration at 50% (IC_50_), calculated via a logarithmic concentration curve. All experiments were performed in triplicate.

### 2.8. Statistical Analysis

Data were presented as mean ± standard error (SD) of three independent experiments. A two-way analysis of variance (ANOVA) was applied to determine the differences in concentration of all polyphenols between samples, and comparisons of the means were carried out using Šídák’s multiple or Tukey’s comparisons tests using GraphPad Prism version 9.0.2. Significance was accepted for *p* < 0.05.

## 3. Results

### 3.1. LC–MS/MS Analysis and Characterization

To evaluate the methods ability to differentiate the molecules of interest from other possible components and interferents present in samples, specificity was checked via repeated injections of analytes into the system, and their retention times were monitored. The presence of the analytes was also confronted and confirmed using the HPLC-DAD method. The LC–MS/MS method we developed achieved great linearity for all analytes (r > 0.998) and was able to correctly characterize and quantify eighteen different phenolic compounds in the different OMWW extracts, even if not all of the analytes were always detectable. The complete quantification results are reported in Table 2. 

The extracts of the first collection (October 2022), CL1 and CF1, were particularly plentiful of oleacein (314.628 ± 19.535 and 227.273 ± 3.974 μg/mg, respectively), tyrosol, and hydroxytyrosol (17.602 ± 0.792, 18.808 ± 0.447 μg/mg, and 55.875 ± 1.511, 71.919 ± 3.943 μg/mg, respectively), with almost comparable quantities in both cultivars. Otherwise, CL1 showed a higher amount of oleocanthalic acid than CF1 (10.628 ± 0.484 and 2.149 ± 0.091 μg/mg, respectively) but lower quantities of verbascoside (1.624 ± 0.035 and 18.182 μg/mg, respectively). Caffeic acid was found in both samples, with comparable concentrations (2.791 ± 0.101 and 2.234 ± 0.112 μg/mg, respectively), whereas the concentrations of oleocanthal (10.628 ± 0.484 (CL1) and 2.149 ± 0.091 μg/mg (CF1)) and 1-acetoxypinoresinol (1.673 ± 0.180 and 0.960 ± 0.109 μg/mg, respectively CL1 and CF1) in CL1 were significantly higher than in CF1. Moreover, CF1 contains 10-fold more oleuropein than CL1 (2.541 ± 0.061 and 0.253 ± 0.001 μg/mg, respectively). Other phenolic compounds were present in both samples in amounts < 1 μg/mg. 

Analyzing the data of the second collection (November 2022) CL2 and CF2 samples, a considerable reduction in all phenolic compounds in comparison to CL1 and CF1 was evident. In fact, almost all polyphenols decreased in concentration (or even disappeared, as for oleuropein in CL and vanillin in CF) in the extract belonging to the second collection (CL2 and CF2) as a result of a decreased amount of polyphenols during the maturation process of olive fruits over the time. In particular, the most evident reduction was detectable for oleacein, the concentration of which had a 100-fold drop in CL OMWW samples (314.628 ± 19.535 vs. 5.896 ± 0.058 μg/mg, mean diff = 308.7, *p* < 0.0001), and a 10-fold decline in CF OMWW samples (227.273 ± 3.974 vs. 23.440 ± 1.257 μg/mg, mean diff = 203.8, *p* < 0.0001). 

### 3.2. Antioxidant/Antiradical Properties

It is well known that EVOO polyphenols display antioxidant and antiradical properties which affect the oxidative stress damage by inhibiting the production of radicals or decreasing the oxidation processes [27]. The high content of nutraceutical polyphenols presented in OMWW prompts the investigation of the nutraceutical properties and, in particular, the antioxidant and antiradical properties. The antiradical properties of CL1, CL2, CF1, and CF2 were tested in vitro by DPPH and ABTS assays, whereas the antioxidant properties were evaluated by the ferric reducing antioxidant power (FRAP) assay. As shown in Table 3, the results revealed that all samples had a good scavenging effect on the DPPH· and ABTS radicals, and the relationship was dose-dependent. The IC_50_ values were lower than 1 mg/mL for all samples, while CF2 presented the best IC_50_ values, ranging the 0.1 mg/mL. Regarding the FRAP assay, the samples of cultivar Frantoio (CF1 and CF2) showed the most interesting activity, in particular CF1 (103.1 ± 5.1 mmol Trolox/g).

### 3.3. Anti-Inflammatory Properties

Inflammation is a fundamental defense mechanism of the organism, sustained by the immune system, that recognizes and eliminates harmful agents and infected cells, promoting tissue repair and restoring body homeostasis with different mechanisms [28,29,30,31]. Polyphenols promote healthy effects, even expressing anti-inflammatory effects with various pathways [32]. EVOO polyphenols are recognized to modulate some inflammatory pathways through a reduction in the expression levels of MMP-9, prostaglandin, and thromboxane (TX) by inhibiting COX-2 and COX-1 enzymes [33,34]. In particular, oleocanthal, oleacein, and hydroxytyrosol induced the inhibition of COX-1 and COX-2 [35,36]; moreover, tyrosol and hydroxytyrosol displayed an inhibitory effect on the arachidonate cascade and the eicosanoid synthesis (PGE2 and LTB4) in cultured macrophages [37]. 

Considering the data reported above, the high content of some of the most representative polyphenols studied for their anti-inflammatory properties, such as oleacein, directed our interest to the investigation of the anti-inflammatory effects of OMWW extracts. CT1, CT2, CF1, and CF2 were evaluated for their ability to inhibit COX enzymes, deeply involved in inflammation cascade.

All the samples were tested at 1 mg/mL, revealing more than 40% inhibition, excluding CL2, which showed about 25% of inhibition. Noteworthy, CL1 and CF1 displayed high inhibition values, especially in COX-2 percentage inhibition, which reached more than 80% (Table 4). Then, COX inhibition percentages for the samples CL1 and CF1 were very promising, especially with respect to COX2 inhibition. The IC_50_ of CF1 and CL1 on the selected enzymes was performed (Table 5). The results revealed a COX-1 IC_50_ ranging 0.5–0.4 mg/mL and a surprising COX-2 IC_50_ value of 0.08 mg/mL for both the extracts. 

## 4. Discussion

In world globalization, the management of different types of waste deriving from food production affects environmental sustainability. For these reasons, the re-valorization of food by-products to produce value-added products is becoming a challenge in the nutraceutical field. 

The olive of *Olea europaea* L. is widely spread in the Mediterranean area, where it accounts for almost 96% of global olive production (FAOSTAT Food and Agriculture Data), and where the majority of EVOO is produced. EVOO is a functional food, rich in exclusive phenolic compounds with diverse health benefits. Unfortunately, EVOO production generates a significant quantity of residues, such as OMWW, olive leaves, and pomace. Nonetheless, a large amount of OMWW remains without application, since only small quantities are used as a source of biomass fuel, and the residual part must be managed with high-cost procedures, accounting environmental concerns. On the other hand, these residues are a precious low-cost starting material from which to produce extracts of dietary polyphenolic molecules that could be used in various nutraceutical fields [38]. However, similarly to EVOO [39], the quality of nutraceutical polyphenols in OMWW differs according to the olive cultivar variety, the technological process of olive oil production, collection time, and climatic conditions [40]. 

For these reasons, in this work, we studied OMWWs produced in a three-phase EVOO extraction system obtained by processing olives of two different Tuscan cultivars, collected in different periods of the EVOO production season, with the aim of evaluating the different polyphenolic content in relation to the cultivars and the olive harvest period. After collection, OMWWs were treated via liquid–liquid extraction in order to recovery dietary polyphenols resulting in dry residue extracts. The resultant OMWW extracts have been analyzed in their polyphenolic profile by the HPLC/MS technique, identifying 18 polyphenols. As expected, a variation in polyphenol composition is clearly noticeable in the analysis of the two different CL and CF OMWW extracts collected at different timepoints. The quantitative analysis of OMWW extracts (Table 2) indicated that all the samples presented an elevated polyphenols content. The samples CF1 and CL1 showed the higher polyphenolic content, probably due to the green maturation level of olive in October. In fact, two states of maturation are evidenced in olive fruits, namely, green and black maturation, that usually correlate with higher and lower polyphenolic content, respectively [41]. During the first collection, the olive fruit were in the green maturation stage, presenting an increased quantity of polyphenols compared to the second collection time (November 2022) in which the olives were black. The polyphenol analysis revealed a high amount of tyrosol, hydroxytyrosol, and phenolic acids for all the samples. Interestingly, an elevated amount of oleacein was quantified in the CL1 and CF1 OMWW extracts (314.628 ± 19.535 and 227.273 ± 3.974 μg/mg, respectively), a very promising polyphenol rarely found in OMWW and generally present in fresh EVOO.

Considering the rich phenolic compound content of these OMWW extracts, their nutraceutical properties were further investigated in order to better comprehend their nutraceutical potential. The DPPH, ABTS, and FRAP assays were used to estimate the antiradical/antioxidant capacity of OMWW extracts (Table 3). The results of the DPPH radical scavenging activity showed that the CF2 extract exhibited the highest antiradical activity, as indicated by the lower IC_50_ value (0.11 mg/mL). Similarly, the data analysis of the ABTS assay showed that the CF2 extract gave the best activity, with an IC_50_ = 0.155 mg/mL. From the results of FRAP, the best antioxidant extracts were CF1 and CF2, presenting a FRAP value ranging from 5 to 6 mmol of Trolox/kg. According to De Marco et al. [23], the phenolic compounds commonly presented in OMWW conferred a strong antioxidant potential. Therefore, the present data clearly showed a positive correlation between the total polyphenols content and the antioxidant/antiradical capacity evaluated by the DPPH, ABTS, and FRAP assays. In fact, our results describe CT1, CT2, CF1, and CF2 as excellent antioxidant extracts, reporting values of inhibition of antioxidant/antiradical significantly lower than the OMWW extracts reported by Belaqziz et al. [42] (IC_50_ DPPH = 16–261 mg/mL) and comparable to the antioxidant properties of other promising OMWW extracts [12]. It is important to demonstrate that the antioxidant activity of these OMWW extracts is significantly improved compared to the antioxidant activity of EVOO extracts [24,43,44], highlighting the nutraceutical potential of these byproducts.

The high content of oleacein and hydroxytyrosol, polyphenols deeply studied for their anti-inflammatory properties [36], suggested the evaluation of OMWW extracts in their ability to inhibit cyclooxygenase enzymes. The inhibitory activity of the two isoforms of cyclooxygenase, COX-1 and COX-2, were evaluated. The COX-1 isoform is a constitutive isoform involved in tissue homeostasis, whereas the COX-2 isoform is an inducible isoform induced by pro-inflammatory stimuli. Surprisingly, all the extracts revealed more than 40% inhibition for both enzymes, except for CL2, which is probably due to the lower polyphenols content and the near absence of oleacein compared to other OMWW extracts. However, CL1 and CF1 reported more than 80% inhibition of COX-2, and these data encouraged further investigations. Therefore, the IC_50_ of CL1 and CF1 were evaluated (Table 5), resulting in significantly low values of COX-2 inhibition for both extracts (0.088 mg/mL for CL1 and 0.082 mg/mL for CF1) and almost 5-fold higher IC_50_ values for COX-1 (0.563 μg/mL for CL1 and 0.418 μg/mL for CF1). The interesting anti-inflammatory values of these extracts are probably due to the high concentration of oleacein and hydroxytyrosol already tested in vitro as potent COX inhibitors (oleacein IC_50_ COX-1 = 0.47 μg/mL and IC_50_ COX-2 = 0.41 μg/mL; hydroxytyrosol IC_50_ COX-1 = 0.02 μg/mL and IC_50_ COX-2 = 0.37 μg/mL) [36]. The high inhibitory potential of these two polyphenols may confer a promising anti-inflammatory ability to CL1 and CF1, rich in oleacein and hydroxytyrosol, probably due to the synergic effects of different phenolic compounds. 

## 5. Conclusions

Nowadays, research has increasingly focused on the valorization and reuse of EVOO by-products because they constitute an environmental concern due to their high production and the related costs of disposal procedures. OMWWs represent one of the most produced byproducts in EVOO production, but at the same time, they are regarded as inexpensive and abundant raw materials rich in bioactive compounds with health-related properties. In this study, we investigated the nutraceutical potential of Tuscan OMWWs produced by two different cultivars of olives (cultivar Leccino (CL) and Frantoio (CF)) and collected in different periods of the olive mill season. The recovery of dietary polyphenolic compounds from OMWWs via liquid–liquid extraction generated dry residue polyphenolic extracts, which were further analyzed via HPLC/MS analysis in order to ascertain their polyphenolic profiles. The OMWW extracts showed high polyphenols content, providing valuable insights into the analysis and variability of OMWW phenolic composition depending on the cultivar and collection time. Overall, oleacein, rarely identified in OMWW, and hydroxytyrosol are the most abundant polyphenols. Furthermore, the investigation of nutraceutical properties revealed potent antioxidant, antiradical, and anti-inflammatory activities. 

The findings underscore the importance of the proper management and valorization of this waste product, which can contribute to the development of sustainable and eco-friendly strategies in the food, pharmaceutical, and cosmetics industries. In fact, the polyphenolic extracts of OMWW, similarly to EVOO polyphenolic extracts, are rich in bioactive compounds and could be used as functional ingredients. The revalorization and reuse of OMWW, recovering its dietary polyphenols, focus on the chance to identify valuable molecules with different biological properties (e.g., antioxidant and anti-inflammatory activities) and are devoted to the further application of these products in food technologies such as in the nutraceutical, cosmeceutical, and pharmaceutical fields. In particular, OMWW polyphenolic extracts, when added in specific formulations as functional ingredients, might increase the nutritional profile of food products, or else they could be innovative natural additives used to improve food properties or active ingredients or additives in cosmetic and pharmaceutical formulations, exploiting their nutraceutical properties. 

Further research and optimization of the extraction methods, such via green extraction, are warranted in order to harness the full potential of OMWW and maximize its value in the nutraceutical industry.

## Figures and Tables

**Table 1:** MS operative parameters. Retention time variations are expressed as mean ± SEM.

Analyte	Retention Time (min)	SRM Transition (Da)	DP (V)	EP (V)	CE (V)	CxP (V)
Tyrosol	7.55 ± 0.01	137.0 →106.0 (Q)	−35	−7.0	−20	−5.0
		137.0 →107.0 (q)			−21	−5.4
		137.0 →119.0 (q)			−20	−5.9
Oleocanthal	10.32 ± 0.01	303.1 →59.0 (Q)	−30	−7.0	−10	−6.0
		303.1 →165.0 (q)			−13	−8.4
		303.1 →182.6 (q)			−13	−9.6
Oleocanthalic Acid	10.40 ± 0.01	319.2 →110.8 (q)	−25	−7.0	−23	−5.3
		319.2 →155.0 (q)			−25	−7.5
		319.2 →199.0 (Q)			−19	−9.8
Hydroxytyrosol	6.49 ± 0.01	152.8 →81.2 (Q)	−90	−7.0	−29	−13.0
		152.8 →93.3 (q)			−30	−11.0
		152.8 →122.6 (q)			−18	−6.0
Caffeic acid	8.04 ± 0.01	179.0 →79.2 (q)	−70	−10.0	−32	−14.0
		179.0 →107.4 (q)			−30	−9.0
		179.0 →134.6 (Q)			−20	−7.0
Syringic acid	8.27 ± 0.01	197.0 →78.2 (Q)	−70	−10.0	−37	−12.0
		197.0 →106.3 (q)			−30	−8.7
		197.0 →123.4 (q)			−35	−6.0
*p*-Coumaric Acid	8.99 ± 0.01	163.0 →65.2 (q)	−70	−7.5	−52	−10.0
		163.0 →93.3 (q)			−40	−5.0
		163.0 →119.2 (Q)			−20	−3.5
Ferulic Acid	9.12 ± 0.01	193.0 →89.2 (Q)	−60	−10.0	−35	−10.0
		193.0 →106.3 (q)			−31	−9.0
		193.0 →149.8 (q)			−30	−8.0
Vanillic Acid	8.12 ± 0.01	167.0 →65.2 (q)	−60	−5.5	−37	−10.0
		167.0 →91.3 (q)			−25	−7.0
		167.0 →108.4 (Q)			−26	−5.0
Vanillin	8.71 ± 0.01	150.8 →92.3 (Q)	−60	−7.0	−27	−7.5
		150.8 →108.4 (q)			−30	−5.0
		150.8 →136.5 (q)			−20	−5.0
Luteolin-7- glucoside	9.22 ± 0.01	447.3 →65.2 (q)	−125	−7.0	−130	−7.0
		447.3 →83.2 (q)			−90	−6.7
		447.3 →132.6 (Q)			−82	−6.3
Verbascoside	8.63 ± 0.01	623.5 →59.0 (q)	−140	−6.0	−116	−7.0
		623.5 →85.2 (Q)			−92	−10.0
		623.5 →133.6 (q)			−110	−6.0
Pinoresinol	10.07 ± 0.10	357.3 →92.2 (Q)	−60	−8.0	−70	−8.0
		357.3 →121.6 (q)			−36	−6.0
		357.3 →136.6 (q)			−45	−6.5
1-Acetoxy pinoresinol	10.11 ± 0.10	415.3 →59.1 (q)	−110	−9.5	−71	−6.0
		415.3 →92.3 (q)			−70	−5.0
		415.3 →136.6 (Q)			−38	−4.7
Oleuropein	9.63 ± 0.20	539.4 →59.1 (Q)	−90	−8.5	−85	−6.5
		539.4 →89.3 (q)			−50	−7
		539.4 →95.3 (q)			−50	−8
Oleacein	9.63 ± 0.01	319.3 →59.1 (Q)	−110	−9.5	−18	−6.4
		319.3 →69.2 (q)			−38	−7.0
		319.3 →95.4 (q)			−36	−8.0
Apigenin-7- glucoside	9.73 ± 0.01	431.2 →63.2 (Q)	−185	−10.0	−101	−9.8
		431.2 →83.1 (q)			−80	−9.3
		431.2 →117.3 (q)			−70	−9.0
Rutin	9.33 ± 0.01	609.4 →65.1 (Q)	−220	−9.0	−160	−10
		609.4 →107.6 (q)			−90	−5.0
		609.4 →108.44 (q)			−90	−5.0
4-Hydroxyphenyl acetic acid (IS)	7.81 ± 0.01	150.8 →79.3 (q)	−70	−7.5	−24	−9.5
		150.8 →105.3 (q)			−24	−4.0
		150.8 →107.4 (Q)			−10	−4.0

**Table 2:** Quantification results of phenolic compounds in analyzed OMWW extracts. Each sample was analyzed in triplicate. Results are expressed as Mean ± SD.

Analyte	CL1 (μg/mg)	CL2 (μg/mg)	CF1 (μg/mg)	CF2 (μg/mg)
Hydroxytyrosol	55.875 ± 1.511	6.821 ± 0.058	71.919 ± 3.943	25.253 ± 1.283
Tyrosol	17.602 ± 0.792	8.119 ± 0.318	18.808 ± 0.447	12.213 ± 1.157
Oleocanthal	2.271 ± 0.071	0.181 ± 0.006	1.059 ± 0.009	0.140 ± 0.006
Oleocanthalic acid	10.628 ± 0.484	3.908 ± 0.151	2.149 ± 0.091	0.120 ± 0.010
Caffeic acid	2.791 ± 0.101	1.453 ± 0.044	2.234 ± 0.112	3.144 ± 0.142
Syringic acid	N/A	N/A	N/A	N/A
*p*-Coumaric acid	0.756 ± 0.028	0.513 ± 0.019	0.428 ± 0.032	0.406 ± 0.016
Ferulic acid	0.108 ± 0.020	0.195 ± 0.018	0.078 ± 0.012	0.040 ± 0.003
Vanillic acid	1.321 ± 0.064	1.356 ± 0.032	0.576 ± 0.010	0.273 ± 0.011
Vanillin	0.018 ± 0.001	0.001 ± 0.001	0.003 ± 0.001	N/A
Verbascoside	1.624 ± 0.035	0.958 ± 0.018	18.182 ± 0.437	0.909 ± 0.035
Luteolin 7 glucoside	0.010 ± 0.001	0.013 ± 0.001	1.115 ± 0.016	0.096 ± 0.004
Pinoresinol	0.391 ± 0.005	0.333 ± 0.009	0.194 ± 0.006	0.194 ± 0.008
Oleuropein	0.253 ± 0.001	N/A	2.541 ± 0.061	0.221 ± 0.006
Oleacein	314.628 ± 19.535	5.896 ± 0.058	227.273 ± 3.974	23.440 ± 1.257
Apigenin 7 glucoside	N/A	N/A	0.025 ± 0.001	0.015 ± 0.001
1-acetoxypinoresinol	1.673 ± 0.180	0.162 ± 0.027	0.960 ± 0.109	1.627 ± 0.045
Rutin	N/A	N/A	0.022 ± 0.004	0.014 ± 0.003

**Table 3:** Antioxidant and antiradical capacities of CL1, CL2, CT1, and CT2 measured by DPPH, ABTS, and FRAP assays.

Sample	DPPH IC_50_ mg/mL	ABTS IC_50_ mg/mL	FRAP mmol Trolox/g
CL1	0.93 ± 0.03 ^a^	0.31 ± 0.015 ^b^	37.75 ± 2.42 ^c^
CL2	0.73 ± 0.01 ^b^	0.43 ± 0.013 ^a^	34.21 ± 0.94 ^c^
CF1	0.50 ± 0.02 ^c^	0.21 ± 0.006 ^c^	103.1 ± 5.1 ^a^
CF2	0.11 ± 0.01 ^d^	0.16 ± 0.002 ^d^	76.36 ± 5.51 ^b^

Values are the average of three determinations ± standard error. Different letters (a, b, c, d) in the same column indicate significant differences between samples (*p* < 0.05).

**Table 4:** Inhibitory effects of CL1, CL2, CF1, and CF2 on COX-1 and COX-2.

Sample	COX-1 % Inhibition	COX-2 % Inhibition
CL1	39.46 ± 0.002	83.6 ± 0.001
CL2	25.30 ± 0.004	24.40 ± 0.002
CF1	48.06 ± 0.002	88.14 ± 0.001
CF2	40.04 ± 0.003	55.80 ± 0.001

Values are the average of three determinations ± standard error.

**Table 5:** IC_50_ values of CL1 and CF1 on COX-1 and COX-2.

Sample	COX-1 IC_50_ (mg/mL)	COX-2 IC_50_ (mg/mL)
CL1	0.563 ± 0.165	0.088 ± 0.008
CF1	0.418 ± 0.145	0.082 ± 0.010

Values are the average of three determinations *±* standard error.

## Data Availability

The datasets generated and analysed during the current study are available from the authors on reasonable request.

## Supplementary Materials

The following supporting information can be downloaded at https://www.mdpi.com/article/10.3390/nu15173746/s1; Table S1: Calibration curves concentrations for each analyte in ESI-MS analysis.
