# Supplementary material for: Olive Mill Wastewater as Source of Polyphenols with Nutraceutical Properties
Source: Nutrients. 2023 Aug 26;15(17):3746. doi: 10.3390/nu15173746 (PMC10489820; doi:10.3390/nu15173746)
Supplement: Supplementary file 1 [file nutrients-15-03746-s001.zip › nutrients-2493581-supplementary.pdf]

Supplementary Materials

Olive mill wastewater as source of polyphenols with nutraceutical properties

Doretta Cuffaro [1,2#], Andrea Bertolini[3#] ,Simone Bertini[1], Claudio Ricci[4], Maria Grazia Cascone[4,5], Serena Danti[4], Alessandro Saba[2,3], Marco Macchia[1,2], Maria Digiacomo[1,2*]

[1] Department of Pharmacy, University of Pisa, via Bonanno 6, 56126, Pisa, Italy. doretta.cuffaro@unipi.it (D.C.), simone.bertini@unipi.it (S.B.), marco.macchia@unipi.it (M.M)
[2] Interdepartmental Research Center "Nutraceuticals and Food for Health", University of Pisa, 56100, Pisa, Italy
[3] Department of Surgery, Medical, Molecular and Critical Area Pathology, University of Pisa, 56126, Pisa, Italy a.bertolini2@student.unisi.it (A.B.), alessandro.saba@unipi.it (A.S.)
[4] Dept. of Civil and Industrial Engineering, University of Pisa, Pisa, Italy; claudio.ricci@unipi.it (C.R.), maria.grazia.cascone@unipi.it (M.G.C.); serena.danti@unipi.it (S.D.)
[5] Dept. of Transl. Res. and New Technologies in Medicine and Surgery, University of Pisa, 56126, Pisa, Italy.

* Corresponding authors: maria.digiacomo@unipi.it (M.D.);
[#] The authors contributed equally to this work.

Table S1. Calibration curves concentrations for each analyte in ESI-MS analysis.

**Analyte Name**	**Range of concentrations in calibration curve (ng/mL)**
Tyrosol, Verbascoside, Rutin	0-3.91-7.81-15.6-31.3-62.5-125-250-500
Oleocanthal, Oleocanthalic acid, Hydroxytyrosol, Ferulic Acid, Vanillic acid, Pinoresinol/1-acetoxypinoresinol, Apigenin-7-glucoside	0-1.95-3.91-7.81-15.6-31.3-62.5-125-250
Caffeic acid, Syringic acid, *p*-Coumaric acid, Vanillin, Luteolin-7-glucoside, Oleuropein	0-0.781-1.56-3.13-6.25-12.5-25-50-100
Oleacein	0-7.81-15.6-31.3-62.5-125-250-500-1000
